# Relationships between *Hha*1 Calpastatin Gene Polymorphism, Growth Performance, and Meat Characteristics of Awassi Sheep

**DOI:** 10.3390/ani9090667

**Published:** 2019-09-07

**Authors:** Khaleel I. Jawasreh, Ahmad H. Al-Amareen, Pauline Y. Aad

**Affiliations:** 1 Department of Animal Production, Jordan University of Science and Technology (JUST), Irbid 22110, Jordan; 2 Livestock Research Directorate, National Agriculture Research center (NARC), Albaqa’a 19381, Jordan; 3 Department of Sciences, Notre Dame University Louaize, Zouk Mosbeh 1211, Lebanon

**Keywords:** Awassi sheep, carcass characteristics, Calpastatin *Hha1* gene, growth trait, meat quality

## Abstract

**Simple Summary:**

The Awassi sheep is a very important breed, and using molecular selection to enhance meat production is important. Following genotyping of Awassi sheep housed at two different research stations in Jordan, three genotypes were identified for the calpastatin *Hha1* gene. Following a fattening trial, the polymorphic calpastatin gene affected final (marketing) body weight and longissimus muscle width. *Hha1* restriction sites found in Calpastatin gene can be used for molecular marker-assisted selection in Awassi for meat purposes.

**Abstract:**

Advances in molecular genetics have allowed the identification of genes that can enhance livestock production. The aim of this study was to investigate possible relationships between the calpastatin (CAST) *Hha*1 gene polymorphisms and growth performance, carcass characteristics, and meat quality in Awassi sheep. A total of 87 blood samples were collected from two-week-old Awassi ram lambs. The amplification of the *CAST Hha*1 gene yielded a fragment of 622 bp. Three *CAST* genotypes were found in Awassi sheep: MM for two fragments (385 bp and 281 bp), MN for three fragments (622 bp, 385 bp, and 281 bp), and NN for only one fragment (622 bp). The M and N allele frequencies of the *CAST Hha*1 genotypes were 0.765 and 0.235, respectively, while the genotypic frequencies of MM, MN, and NN were 0.586, 0.356, and 0.057, respectively. Based on *CAST* *Hha*1 gene polymorphisms, three groups of lambs (MM: *n* = 8; MN: *n* = 6; and NN: *n* = 3 genotypes) were subjected to a fattening period of 70 days to investigate growth performance and meat characteristics. Only the final body weight and longissimus muscle width were significantly different between the three genotypes, while no significant differences were detected in any other carcass characteristics and meat quality parameters. In this study, new variants were observed in CAST using the *Hha*1 restriction site, potentially assisting in Awassi sheep breeding and selection programs to improve final body weight and longissimus muscle width.

## 1. Introduction

Awassi sheep is a multi-purpose, fat-tailed sheep breed [1], predominantly found in Jordan and the Middle East [1]. This breed is well-adapted to harsh environmental conditions [2], and is used for both meat and milk production [1]. High variation has been observed in the growth performance of Awassi sheep [1,2]. However, in recent years, substantial advances have been made through the application of molecular genetics in the identification of different chromosomal regions and loci that affect traits of interest in livestock production [3]. This has provided opportunities to launch genetic improvement programs in livestock by the direct selection of genes or genomic regions that affect economically-profitable traits through marker-assisted selection and gene introgression [4]. Growth traits and carcass characteristics are controlled by multiple genes. Calpastatin (*CAST*), for example, plays a significant role in growth traits and carcass characteristics [5]. *CAST* works by inhibiting protease enzymes that degrade muscle protein, and influences meat production, tenderness, and quality [6]. The effects of *CAST* gene polymorphism on growth performance, carcass characteristics, and meat quality have not been fully investigated in Awassi sheep. Therefore, this study was conducted to investigate possible relationships between the *CAST Hha*1 gene polymorphisms and growth performance, carcass characteristics, and meat quality in Awassi sheep.

## 2. Materials and Methods

All procedures used in this study were approved by the Animal Care and Use Committee (ACUC) at Jordan University of Science and Technology, Jordan, approval Number 14/5/5/145.

### 2.1. Genotyping and PCR Analysis

A total of 87 blood samples were collected from two-week-old Awassi ram lambs housed at two experimental sheep breeding stations: the National Agricultural Research Center (NARC), Al Khanasry Research Station (42 ram lambs), and the Jordan University of Science and Technology Center (JUST) for Research and Production (45 ram lambs), both located in the northern part of Jordan. Blood samples were stored at 20 °C until DNA extraction was performed using the E.Z.N.A Blood DNA kit (Omega Bio-tek, United States), and the quality of isolated DNA was tested using 1.5% agarose gel electrophoresis.

Polymerase Chain Reaction- Restricted Fragment Length Polymorphism (PCR-RFLP) genotyping was used as previously described [7,8] to find new possible polymorphisms in the region between exons 61 bp 1C, 88 bp 1D, and 473 bp of the *CAST* gene previously studied [8]. A 622 bp amplicon was amplified using forward 5′-TGGGGCCCAATGACGCCATCGATG-3′ and reverse 5′- GGTGGAGCAGCACTTCTGATCACC-3′ primers on the Life Pro Thermal Cycler (Hangzhou Bioer Technology Co., Hangzhou, China). The optimum annealing temperature for PCR was determined empirically using gradient PCR, using reaction mix and conditions as described previously [7,8]. Briefly, initial denaturation was conducted at 95 °C for 3 min, followed by 35 cycles of annealing at 63 °C for 50 s, polymerization at 72 °C for 60 s, and a final extension at 72 °C for 10 min. Then 10 μL of the PCR product was digested with Hha1 restriction enzyme at 37 °C for 12 h, and resolved on 2% agarose gel, as described [7]. One PCR product from each different genotype of the CAST gene was purified and sequenced in both directions to confirm the detected genotypes, using 3730 × l DNA Analyzer and Big Dye Terminator v3.1 Cycle Sequencing Kit (Thermo Fisher Scientific, United States).

### 2.2. Fattening Trial and Meat Evaluation

Depending on the detected *CAST* gene genotypes, three groups of lambs (MM: *n* = 8; MN: *n* = 6; and NN: *n* = 3) were subjected to a fattening period of 70 days to investigate growth performance and meat characteristics. During the fattening period, lambs were housed individually in shaded pens (1.5 m × 1.75 m). All lambs were introduced slowly to the fattening diet, which contained 16% crude protein and 2.78 Mcal metabolizable energy/kg [9], composed of 15% soybean, 61.4% barley, 21% wheat straw, 1.5% salt, 0.1% limestone, and 0.1% minerals and vitamins. The diet as a total mixed ration (TMR) and fresh drinking water were offered ad libitum. Fresh feed and leftovers were weighed daily for the calculation of feed intake and feed efficiency. Live body weight was recorded biweekly.

At the end of the fattening period, all lambs were slaughtered for the evaluation of carcass traits and meat quality, as previously described [10,11,12]. Briefly, carcasses were divided into four parts, namely shoulder, rack, loin, and leg cuts, in addition to the fat tail and the rib-eye area, fat depth, tissue depth, rib fat depth, eye muscle width, eye muscle depth, eye muscle area, and leg fat depth, measured as previously described [10,12]. The right leg was dissected to determine muscle, bone, subcutaneous fat, and intermuscular fat components. Longissimus muscles were excised from the right side of loin cuts, cleaned from all subcutaneous fat, vacuum-packaged, and frozen at −20 °C for meat quality measurements, as described [13]. Meat quality parameters were Warner–Bratzler shear force values for cooked meat samples, water holding capacity, cooking loss, and color coordinates (whiteness, redness, yellowness) [10,12,13].

### 2.3. Statistical Analysis

Data were analyzed using the general linear model (GLM) of SAS (version 8.1, SAS Inc., Cary, NC, USA). The genotype was inserted as the only fixed effect in the model. The initial body weight was inserted as a covariate for the following traits: final body weight, fasting live weight, hot carcass weight, cold carcass weight, dressing percentage, and average daily gain. Dry matter intake, feed conversion ratio, carcass cut, dissected leg, non-carcass components, and meat quality parameters were also analyzed using the GLM procedure of SAS. Least square means of the GLM procedure of SAS was used to further identify significant differences among the means. Significant differences were considered at *p* ≤ 0.05.

## 3. Results and Discussions

### 3.1. Genotyping of Awassi Ram Lambs for the *CAST* Gene

**Figure 1:**
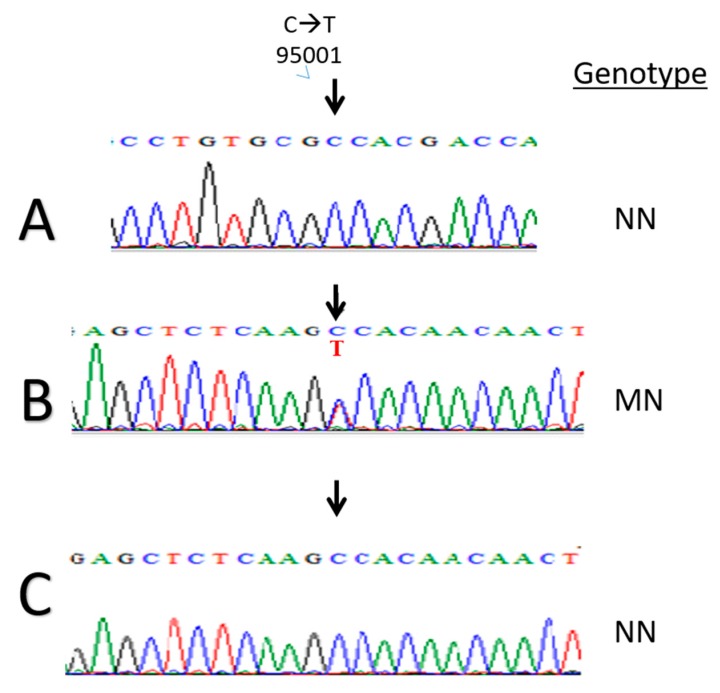
The calpastatin (*CAST*) gene sequencing of animals with the various genotypes. The arrow indicates the site of the new mutation from C to T, not registered in NCBI, aligning to position 95,001 bp of the ovine calpastatin gene (accession number 443364). (**A**) NN or CC genotype variant. (**B**) MN or CT genotype variant, where two sequencing bands appear. (**C**) the same as A.

**Figure 2:**
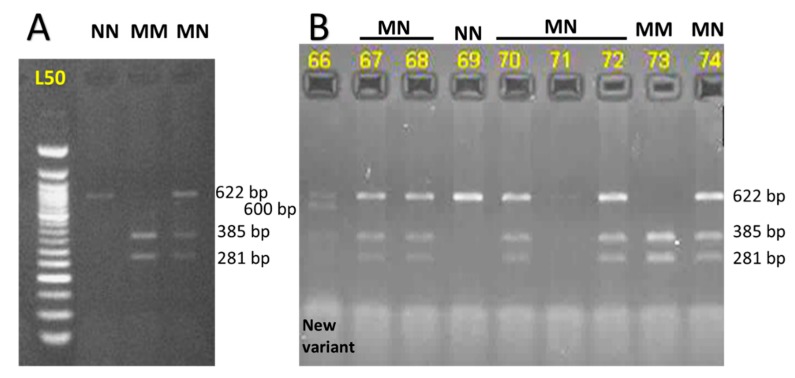
Restriction digestion pattern for calpastatin gene with *Hha*1. (**A**) RFLP analysis of the PCR product of the *CAST* gene following digestion with the Hha1 restriction enzyme, where the different genotypes were sized. Lane L50: 50 bp DNA ladder (Fermentas); Lane NN: genotype NN of one fragment (622 bp band); lane MM: genotype MM two fragments (385 bp and 281 bp band); lane MN: genotype MN of three fragments (622 bp, 385 bp, and 281 bp band). (**B**) RFLP analysis of the various animals using Hha1 restriction endonuclease. Genotypes of the various animals (NN, MM, or MN) are indicated above the loading lane. A rare variant in animal 66 is shown.

The *Hha*1 calpastatin gene 622 bp PCR fragment, as previously measured [8] by the ladder on a 2% agarose gel, was sequenced following purification (Figure 1). Three genotypes were found in Awassi sheep as shown in Figure 2A: MM or TT genotypes of two fragments (385 bp and 281 bp), MN or CT genotype of three fragments (622 bp, 385 bp, 281 bp), and NN or CC genotype of only one fragment (622 bp), by Hha1 restriction enzyme analysis. The M or T and N or C allele frequencies of the *CAST Hha*1 genotypes were 0.765 and 0.235, respectively, while the genotypic frequencies of NN, MN, and MM were 0.057, 0.356 and 0.586, respectively. To our knowledge, there are no reports that have investigated the different restriction sites found in the ovine *CAST* gene using the *Hha*1 variant, as shown on animal 66 (Figure 2B), which showed a rare variant where a 600 bp fragment was observed.

### 3.2. Evaluation of Awassi Fattening Response to Various Genotypes of the *CAST* Gene

Following a 70 day fattening period for the Awassi ram lambs, significant differences were observed between *CAST* genotypes for final body weight (Table 1). The MM genotype had the highest body weight, followed by MN and NN. Fasting body weight, average daily gain, dry matter intake, and feed conversion ratio were non-significantly affected by the *Hha*1 genotypes. However, overall weight gain (data not shown) was greater in MM, which was significantly different from either MN or NN genotypes. These results are contradictory to previous findings, where a strong association between the calpastatin gene and body weight was reported in male sheep in Indonesia, with the MN showing higher (*p* < 0.017) body weights than NN genotype ram lambs [14]. Jawasreh et al. [8] found that live weight and average daily gain were higher in Awassi ram lambs that carry the MN genotype when compared to the MM genotype. The size of skeletal muscles is largely dependent on the balance between the rate of degradation and the rate of synthesis, as well as the inhibitory effect of the calpastatin for the calpains [15]. This may result in a decreased degradation rate of protein and an increased rate of protein synthesis in skeletal muscles. The identified polymorphisms for the calpastatin gene, using *Hha*1, show rare occurrence, and might contribute less than other polymorphic forms using *Msp*I [8].

The three *CAST* genotypes did not (*p* > 0.05) influence hot carcass weight, cold carcass weight, dressing percentage, non-carcass components (heart, liver, spleen, kidney, kidney fat, mesenteric fat, testis, fat tail, lung, and trachea), and carcass cuts weights (shoulders, leg, loin) (Table 1). Dissected leg cuts were also comparable (*p* > 0.05) in their intermuscular fat, subcutaneous fat, total fat, total lean, and total bone. No significant differences were found in longissimus muscle weights among the three genotypes (Table 1). With Awassi sheep, the high population variability, alongside the rarity of the *CAST* mutations, might actually contribute to the discrepancy between ours and others results with regard to the contribution of the *CAST* gene in muscling.

The longissimus muscle widths differed slightly (*p* = 0.0518) between the genotypes with the highest width, found in the MN genotype followed by MM (Table 2). The longissimus muscle width in the MM and MN genotypes were both significantly different from the NN genotype (*p* = 0.05). The longissimus muscle area, tissue depth, fat depth, rib fat depth, fat thickness, muscle area, width, and depth were comparable (*p* > 0.05) among the different *CAST* genotypes (Table 2). The rarity of the genotypes analyzed in this study partly contributed to the low number of animals analyzed, and thus a larger scale study might show higher levels of significance for the effect of the calpastatin genotypes on the final body weights and longissimus muscle width in Awassi lambs. Jawasreh et al. [8] found that Awassi sheep that carried the MM genotype had a higher total bone weight than MN genotypes, while lambs that carry the MN genotype had a higher (*p* ≤ 0.05) meat-to-bone ratio than the MM genotype. In Kivircik sheep, Yilmaz et al. [16] reported significant differences among calpastatin genotypes in back fat thickness and skin and backfat thickness values of the loin eye muscle. In addition, Yilmaz et al. [16] also found that Kivircik lambs with MN and MM genotype had lower carcass fat than those of the NN genotype. In a study carried out on Afshari lambs, no correlation between calpastatin genotypes and carcass traits was reported [17]. Greguła-Kania [18] found that the longest muscle (M. longissimus lumborum) and fat thickness were not affected by *CAST* genotypes in lambs at 80 and 120 days of age. Also, there was no association between different *CAST* gene variants and carcass weight or dressing percentage of thin-tail sheep [19]. The lack of changes in the fat depth and thickness in Awassi sheep could be due to the way Awassi sheep mobilize their fat reserves to their tails rather than to their intra- or intermuscular spaces. Altogether, these results show the breed distinction in their response the various *CAST* gene mutations, and thus the need to investigate the *CAST* gene with other restriction enzymes to identify further mutations and explore their significance.

Meat quality characteristics in each *CAST* genotype in Awassi ram lambs are shown in Table 3. All meat quality parameters were similar (*p* > 0.05) among the MM, MN and NN CAST genotypes. These results are in agreement with Dagong et al. [19], who reported a non-significant difference among calpastatin genotypes in meat tenderness, pH, water holding capacity, and cooking loss of thin-tail sheep. Inconsistent with our findings, many studies revealed a significant effect of the variation in the calpastatin gene on meat quality in Awassi sheep [8]. The size of skeletal muscle fibers, the muscle protein synthesis rate, and the muscle protein degradation rate are among the characteristics that are the most affected by calpastatin gene polymorphism in pigs [20]. With the calpain system playing an important role in muscle fiber growth [20,21], and with the calpain system activity decreasing with the increase in CAST gene activity [20], the calpain activity may play an imperative role that is necessary for cell proliferation, myoblast fusion, fibers growth, and fiber numbers [22]. The calpain system influence can also be observed in fiber numbers, by modulating myoblast fusion and changing the myoblast proliferation rate [23].

## 4. Conclusion

In this study, new variants were observed in *CAST* using the *Hha*1 restriction site, with the NN genotype of the calpstatin gene presenting a low frequency in the Awassi flock; this accounts for the low number of samples analyzed, a major limit of this research. The MM genotype had the highest final body weight, and MM and MN genotypes had a tendency to show the highest longissimus muscle width. For further analysis of a larger population, a potential impediment without marker-assisted selection for such genotypes and the associated costs, our results suggest a potential for the calpastatin to be a useful tool in some, but not all genotypes. This is further strengthened by the lack of association observed between the three detected genotypes and the other growth performance and meat characteristics of Awassi lambs. With calpastatin contributing to improved final body weight, this gene of the MM genotypes is best used in marker-assisted molecular selection programs designed for improving the final body weight and longissimus muscle width in Awassi sheep.

## Figures and Tables

**Table 1:** Least-square means (± standard error (SE)) for growth performance and carcass components, according to the different genotypes of the *CAST*/*Hha*1 gene in Awassi ram lambs.

Parameters	*CAST*/*Hha*1 Genotype			*p*-Value
	MM	MN	NN	
* Initial body weight (kg)	22.5 ± 2.77	24.3 ± 2.40	20.3 ± 3.92	0.6891
Final body weight (kg)	40.2 ± 1.68 ^a^	38.2 ± 1.47 ^a^	36.9 ± 1.42 ^b^	0.0456
Fasting body weight (kg)	39.1 ± 1.60	37.7 ± 1.40	36.2 ± 2.30	0.5850
Average daily gain (kg)	0.302 ± 0.03	0.266 ± 0.03	0.245 ± 0.04	0.5036
Dry matter intake (kg)	62.6 ± 6.01	63.9 ± 5.20	58.6 ± 8.50	0.8673
Feed conversion ratio (kg feed/kg gain)	3.68 ± 0.32	4.31 ± 0.28	4.55 ± 0.45	0.2353
Hot carcass weight (kg)	18.6 ± 0.92	19.4 ± 0.94	17.4 ± 1.19	0.4628
Cold carcass weight (kg)	18.2 ± 0.91	19.1 ± 0.93	16.9 ± 1.18	0.4250
Dressing%	50.7 ± 1.25	50.8 ± 1.25	48.9 ± 1.61	0.3141
Legs (kg)	5.49 ± 0.61	6.43 ± 0.61	5.02 ± 0.79	0.3605
Loins (kg)	1.69 ± 0.27	1.98 ± 0.27	1.72 ± 0.35	0.7222
Shoulders and racks (kg)	7.94 ± 0.89	9.15 ± 0.89	7.06 ± 1.15	0.3677
Fat tail (kg)	1.76 ± 0.46	2.29 ± 0.46	1.48 ± 0.59	0.5347
Heart weight (kg)	0.13 ± 0.01	0.15 ± 0.01	0.13 ± 0.02	0.5390
Liver weight (kg)	0.60 ± 0.06	0.62 ± 0.06	0.53 ± 0.08	0.6478
Lungs and trachea weight (kg)	0.55 ± 0.06	0.53 ± 0.06	0.44 ± 0.07	0.4877
Spleen weigh (kg)	0.06 ± 0.01	0.07 ± 0.01	0.05 ± 0.01	0.3874
Kidney weight (kg)	0.09 ± 0.01	0.11 ± 0.01	0.11 ± 0.01	0.4299
Kidney fat weight (kg)	0.13 ± 0.03	0.19 ± 0.03	0.15 ± 0.04	0.3939
Mesenteric fat weight (kg)	0.22 ± 0.07	0.38 ± 0.07	0.25 ± 0.09	0.2990
Testes weight (kg)	0.19 ± 0.03	0.18 ± 0.03	0.11 ± 0.04	0.3665
Loin (kg)	0.78 ± 0.13	0.96 ± 0.13	0.83 ± 0.17	0.6094
Longissimus weight (kg)	0.22 ± 0.03	0.26 ± 0.03	0.20 ± 0.04	0.6033
Leg weight (kg)	2.78 ± 0.29	3.17 ± 0.29	2.42 ± 0.38	0.3239
Total lean (kg)	1.60 ± 0.15	1.80 ± 0.15	1.30 ± 0.20	0.2371
Intermuscular fat (kg)	0.066 ± 0.01	0.092 ± 0.01	0.066 ± 0.01	0.1419
Subcutaneous fat (kg)	0.39 ± 0.07	0.51 ± 0.07	0.35 ± 0.09	0.3902
Total bone (kg)	0.57 ± 0.06	0.61 ± 0.06	0.48 ± 0.08	0.4387

* Initial weight was inserted as covariate for the following traits: final body weight, fasting live weight, hot carcass weight, cold carcass weight, dressing percentage, and average daily gain. ^a,b^ Means with different superscripts differ significantly (*p* ≤ 0.05).

**Table 2:** Least-square means (± SE) for longissimus linear dimensions and fat measurements, according to different genotypes of the *CAST Hha*1 gene in Awassi ram lambs.

Parameters	*CAST*/*Hha*1 Genotype			*p*-Value
	MM	MN	NN	
Tissue depth (mm)	14.4 ± 1.53	18.0 ± 1.53	14.6 ± 1.98	0.2487
Rib fat depth (mm)	6.20 ± 1.04	8.30 ± 1.04	6.50 ± 1.34	0.3584
Fat depth (mm)	4.30 ± 1.00	6.20 ± 1.00	4.50 ± 1.29	0.3952
Fat thickness (mm)	10.2 ± 1.80	13.0 ± 1.80	9.5 ± 2.3	0.4307
Longissimus area (cm^2^)	15.2 ± 1.51	16.3 ± 1.51	11.8 ± 1.95	0.2210
Longissimus width (mm)	63.6 ± 2.54 ^a^	66.3 ± 2.54 ^a^	55.8 ± 3.28 ^b^	0.0518
Longissimus depth (mm)	27.8 ± 3.15	33.6 ± 3.15	24.3 ± 4.07	0.2179

^a,b^ Row means where different superscripts differ significantly (*p* ≤ 0.05).

**Table 3:** Least-square means (± SE) for meat quality characteristics, according to different genotypes of the *CAST Hha*1 gene in Awassi ram lambs.

Parameters	*CAST*/*Hha*1 Genotype			*p*-Value
	MM	MN	NN	
pH	5.71 ± 0.02	5.75 ± 0.02	5.72 ± 0.03	0.5932
Cooking loss (%)	47.2 ± 0.68	46.5 ± 0.68	47.3 ± 0.88	0.6989
Water holding capacity (%)	38.3 ± 1.69	36.9 ± 1.69	38.2 ± 2.18	0.8171
Shear force (kg/cm^2^)	5.21 ± 1.13	5.87 ± 1.13	5.28 ± 1.46	0.9077
Color coordinates				
Whiteness	34.08 ± 1.42	34.76 ± 1.42	37.99 ± 1.84	0.2694
Redness	3.06 ± 0.42	3.57 ± 0.42	2.19 ± 0.54	0.1878
Yellowness	18.78 ± 0.82	17.66 ± 0.82	19.64 ± 1.06	0.3523
